# Development of a biodegradable polycaprolactone film incorporated with an antimicrobial agent via an extrusion process

**DOI:** 10.1038/s41598-019-56757-5

**Published:** 2019-12-27

**Authors:** Ji Sou Lyu, Jung-Soo Lee, Jaejoon Han

**Affiliations:** 1 Department of Biotechnology, College of Life Sciences and Biotechnology, Korea University, Seoul, 136-701 Republic of Korea; 2 Department of Food Bioscience and Technology, College of Life Sciences and Biotechnology, Korea University, Seoul, 02841 Republic of Korea

**Keywords:** Chemistry, Materials science

## Abstract

In the present study, polycaprolactone (PCL) composite films incorporated with various concentrations of grapefruit seed extract (GSE) as an antimicrobial agent were prepared using a twin-screw extruder. Physical characteristics as well as antimicrobial properties of the PCL/GSE composite films were analyzed. The results showed that the surface color of the films gradually changed with increasing GSE concentration. Fourier transform infrared spectra indicated no significant structural changes such as chemical bond formation between PCL and GSE. Thermal properties were slightly affected due to GSE incorporation. Crystallinity of the composite films decreased as the amount of GSE increased. *In vitro* analysis indicated that the antimicrobial activity of the PCL/GSE composite films increased as the GSE concentration increased, with a 5% concentration showing the strongest inhibitory activity against *Listeria monocytogenes*, with 5.8-log reduction in bacterial count. Application testing of the films was carried out for cheese packaging, and biodegradation of the samples was assessed via soil burial testing. Our findings confirmed the potential use of PCL/GSE composite films as biodegradable food packaging material with antimicrobial activity.

## Introduction

Various polymers such as polyethylene terephthalate (PET), polypropylene (PP), and polystyrene (PS) have been widely used as common packaging materials due to their low cost and high processability^1^. However, excessive use of these films has led to serious environmental problems due to their non-biodegradability. There has been increasing global awareness regarding environmental pollution problems caused by these non-degradable plastic wastes^2^. Therefore, efforts to develop eco-friendly biodegradable films are needed.

In the development of antimicrobial packaging, biodegradability is required beyond just functional aspects. Accordingly, we developed biodegradable antimicrobial packaging films using polycaprolactone (PCL) and grapefruit seed extract (GSE) as polymeric material and antimicrobial agent, respectively. Many other researchers who studied the development of biodegradable composite films claimed that the raw materials used were biodegradable substances, so the composite films produced by mixing them were also biodegradable. On the other hand, we tried to suggest the actual degree of the biodegradability of the developed films through changes in weight loss in the films during soil burial tests.

Among various biodegradable polymers, PCL is a hydrophobic semi-crystalline linear aliphatic polyester with a low melting point (approximately 60 °C) and a glass transition temperature (approximately −60 °C) which allow easy processing^3^. PCL is reported to be decomposed by extracellular depolymerase or in various natural environments^4^. Therefore, PCL has attracted attention as a biodegradable packaging material that can replace non-biodegradable materials. Despite its many advantages, only few studies have explored PCL as a biodegradable material for food packaging due to its low thermal stability. The low melting point of PCL allows easy processing but also limits its application^5,6^; however, it could be appropriate as packaging material for refrigerated foods such as fresh salads and cheeses.

Microbial growth on food surfaces is a major cause of food deterioration^7^. Therefore, antimicrobial packaging films are fabricated by incorporating antimicrobial agents into the polymeric materials or blending them during polymer processing stage^8^. One of the natural antimicrobial substances, GSE has been widely used to retard or reduce bacterial growth for extending shelf-life of foods^9^. The antimicrobial activity of GSE has been attributed to the antioxidant activity of bioactive flavonoids such as naringin, naringenin, hesperidin, and hesperitin. The organic acids, including ascorbic acid and citric acid also involved in antimicrobial activity of the GSE^10,11^. Among the components of the GSE, naringin is a major constituent found in the seed of ripe grapefruits and is associated with a bitter taste^12^. It is water-soluble and is thus used in a wide range of applications; further, it is nontoxic even when consumed in excessive amounts. GSE is heat-stable up to 120 °C^13^. Therefore, it can be incorporated with various polymeric packaging materials without thermal decomposition under manufacturing conditions below 120 °C.

In the film manufacturing methods, solution casting technique has been widely used in preparation of packaging materials at laboratory scale because of its ease and simplicity. However, solution casting technique is normally not practicable for commercial film production. This type of film production is difficult to scale-up and it includes several steps (solubilization, casting, and drying), which causes relatively long processing time^14^. Whereas, extrusion technique can be performed as a continuous unit operation with control of temperature, size, shape, and moisture^15^. Extrusion technique comparing to solution casting technique, it provides more structured film and allows better dispersion of active compounds in the polymers^16^. Therefore, extrusion technique is usually preferred in industrial application^17^. Nevertheless, there have been few studies on the preparation of biodegradable antimicrobial films using extrusion.

Thus, the objectives of this study were to (1) produce biodegradable antimicrobial PCL-based film incorporated with GSE using a twin-screw extruder; (2) determine structural, colorimetric, thermal, and mechanical properties of the films; (3) investigate *in vitro* and *in vivo* (real food system, cheddar cheese packaging) antimicrobial activity of the films against growth of *Listeria monocytogenes*; and (4) examine biodegradability of the films according to soil burial test.

## Results and Discussion

### Characterization of PCL/GSE composite films

#### Fourier transform infrared (FT-IR) spectroscopy

**Figure 1:**
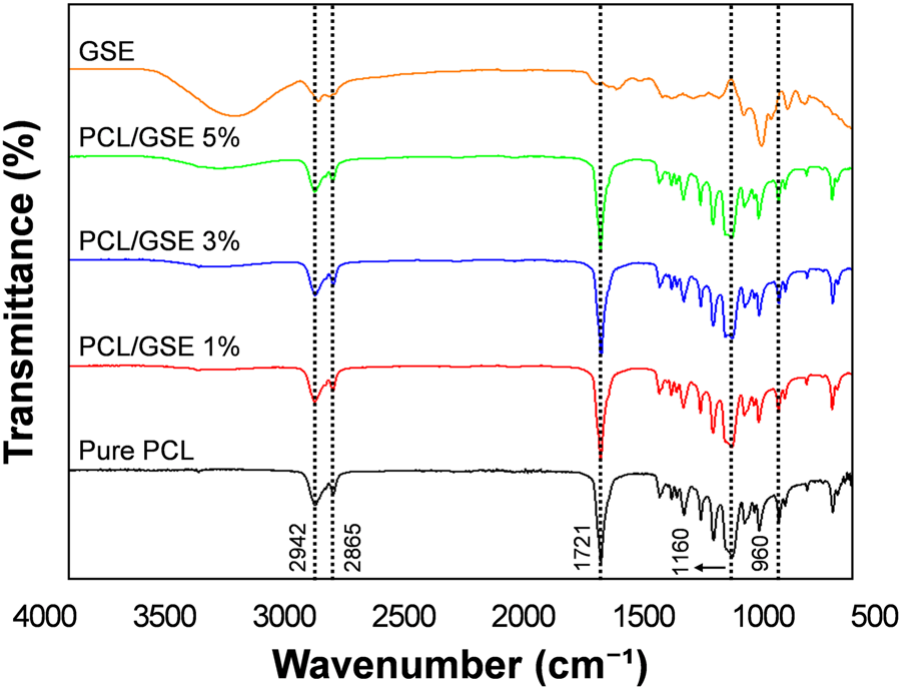
FT-IR spectra of pure PCL, GSE, and PCL/GSE composite films. PCL: polycaprolactone; GSE: grapefruit seed extract.

FT-IR spectroscopy analysis was performed to investigate chemical interactions between PCL and GSE. FTIR spectra of pure PCL and PCL/GSE composite films are shown in Fig. 1. In the spectrum of pure PCL film, weak peaks observed at 2942 cm^−1^ and 2865 cm^−1^ corresponded to asymmetric elongation of the methylene-oxygen (CH_2_‒O) and symmetric methylene groups (CH_2_‒), respectively^18^. The sharp and strong peak at 1721 cm^−1^ represents vibration of –C=O bonds^19^. In addition, representative peaks at 1166 cm^−1^ (C–O–C bond) and 960 cm^−1^ (stretching of the oxime bond) were also detected. In GSE spectrum, a broad absorption band observed at 3600–3100 cm^−1^ is attributed to typical OH vibrations of phenolic/aromatic compounds^20^. Peaks in the spectrum of the pure PCL film were similar to those of the PCL/GSE composite films. However, a broad peak at 3300 cm^−1^ was absent in the spectrum of pure PCL film but present in the spectra of the PCL/GSE composite films, appearing gradually as the GSE content increased; this may be due to the flavonoid group in GSE.

In brief, the FT-IR peaks in the PCL/GSE composite films spectra were similar to those in the spectrum of the pure PCL film, suggesting that there were no significant structural changes due to the addition of GSE in PCL matrix, such as new chemical bond formation between the PCL chains and GSE.

#### Color

Surface color values of the pure PCL and PCL/GSE composite films is presented in Table 1. The L^*^ and a^*^ values of the PCL/GSE composite films were significantly decreased (*p* ≤ 0.05), the b^*^ values were significantly increased (*p* ≤ 0.05), according to the addition of GSE. These results suggested that the PCL/GSE composite films had more intense dark, green, and yellow tones compared with the pure PCL film, which was due to the opacity and yellowish tint of the GSE. Similar results have been reported with other GSE composite films based on agar^8^ and carrageenan^21^.

**Table 1:** Color parameters of the pure PCL and PCL/GSE composite films.

Composite	Color parameters			
	L^*^	a^*^	b^*^	ΔE^*^
Pure PCL	94.40 ± 0.06^D^	3.86 ± 0.04^D^	1.16 ± 0.06^A^	2.72 ± 0.06^A^
PCL/GSE 1%	93.59 ± 0.16^C^	3.78 ± 0.04^C^	2.76 ± 0.16^B^	4.07 ± 0.18^B^
PCL/GSE 3%	91.08 ± 0.06^B^	3.70 ± 0.02^B^	6.83 ± 0.24^C^	8.61 ± 0.21^C^
PCL/GSE 5%	90.24 ± 0.17^A^	3.33 ± 0.02^A^	9.65 ± 0.14^D^	11.32 ± 0.09^D^

PCL: polycaprolactone; GSE: grapefruit seed extract.

Data are expressed as the mean ± standard deviation of five replicates.

L^*^: lightness; a^*^: redness; b^*^: yellowness; ΔE^*^: total color difference.

The different uppercase letters (A–D) in the same column indicate a significant difference (*p* ≤ 0.05), as assessed by Duncan’s multiple range test.

#### Differential scanning calorimetry (DSC)

DSC analysis was performed to investigate the thermal properties of the pure PCL and PCL/GSE composite films. The main thermal parameters, including the melting temperature (*T*_m_), the enthalpy of melting *(∆H*_m_), and the degree of crystallinity (*X*_c_) are shown in Table 2. All films indicated one clear melting peak at slightly above 60 °C. The pure PCL film prepared in the present study showed a *T*_m_ of 62.93 °C and an *∆H*_m_ of 67.72 ± 0.25 J/g, closely analogous to values reported previously^22^. The thermal behaviors of the PCL/GSE composite films were similar to that of the pure PCL film, but the *T*_m_ and *∆H*_m_ of the composites were slightly but significantly decreased (*p* ≤ 0.05) to 60.91 °C and 59.76 ± 1.93 J/g in the PCL/GSE 5% composite film group. In addition, the crystallinity percentage (*X*_c_%) of the PCL/GSE composites film was calculated and found to gradually decrease to 43.91% compared with that of pure PCL film (49.76%) as the GSE content increased.

**Table 2:** Thermal properties of the pure PCL and PCL/GSE composite films.

Composite	Thermal properties		
	*T*_m_ (°C)	Δ*H*_m_ (J/g)	*X*_c_ (%)
Pure PCL	62.93^C^	67.72^C^	49.76^C^
PCL/GSE 1%	61.77^B^	64.58^B^	47.45^B^
PCL/GSE 3%	61.33^AB^	61.66^A^	45.31^A^
PCL/GSE 5%	60.91^A^	59.76^A^	43.91^A^

PCL: polycaprolactone; GSE: grapefruit seed extract.

Data are expressed as the mean of three replicates.

*T*_m_: melting temperature; Δ*H*_m_: the enthalpy of melting; *X*_c_: the degree of crystallinity.

The different uppercase letters (A–C) in the same column indicate a significant difference (*p* ≤ 0.05), as assessed by Duncan’s multiple range test.

#### Thermogravimetric analysis (TGA)

**Figure 2:**
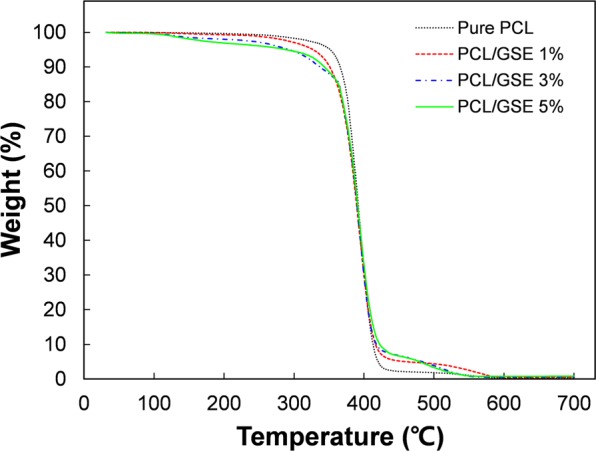
TGA graphs of pure PCL and PCL/GSE composite films. PCL: polycaprolactone; GSE: grapefruit seed extract.

The thermogravimetric analysis results of the PCL films incorporated with various concentrations of GSE are shown in Fig. 2. Pure PCL film showed a simple decomposition profile with a single transition temperature. Incorporation of GSE slightly affected the thermal stability of the pure PCL film. All composites showed a 3-step decomposition. The first step was observed at approximately 90 °C, which may have been due to water evaporation inside the PCL/GSE composite films. The second and third steps of degradation could have been due to the decomposition of glycerol and GSE in PCL polymer, respectively. The second stage was recorded at approximately 120–200 °C, and can be attributed to the decomposition of glycerol. The main decomposition was observed at approximately 400 °C and may have been due to the polymeric degradation of PCL. Incorporation of GSE into the PCL matrix appeared to decrease thermal stability but the decrease was not significant (*p* > 0.05). Previous reports regarding PCL composite films have shown similar results^23,24^. Thus, thermal stability of the PCL/GSE composite films was only slightly affected due to GSE incorporation, implying that GSE addition did not significantly alter (*p* > 0.05) thermal stability of the PCL-based films.

#### X-ray diffraction (XRD)

**Figure 3:**
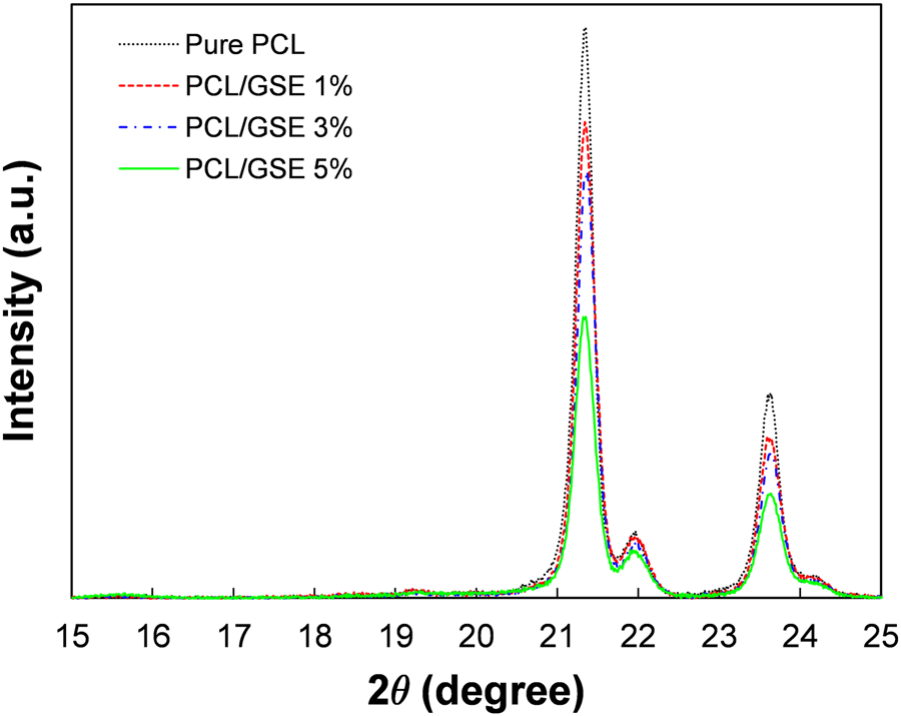
XRD patterns of pure PCL and PCL/GSE composite films. PCL: polycaprolactone; GSE: grapefruit seed extract.

The XRD patterns at 2θ ranging from 15 to 25° of the pure PCL and PCL/GSE composite films are presented in Fig. 3. The pure PCL exhibited two main characteristic peaks at a Bragg angle of 2θ = 21.4 and 23.8° which are due to the (110) and (200) reflections, respectively^25^. Further, it indicates the semi-crystalline property of the PCL polymer^26^. Though the diffraction patterns of all the PCL/GSE composite films were similar to that of the pure PCL, the peak intensities were lowered by increasing amount of GSE, indicating a loss of crystallinity which is in accordance with the above mentioned the degree of crystallinity (*X*_c_) data in the DSC thermogram. Reduction in crystallinity indicates an extension of amorphous regions, and this phenomenon further affected the biodegradability of the pure PCL and its composite films.

#### Mechanical properties

The mechanical properties including tensile strength and elongation at break of the pure PCL and PCL/GSE composite films were investigated to assess the effect of the GSE in the PCL/GSE composite films (Table 3). The general mechanical properties of the pure PCL film are known to be characterized by extremely high elongation at break and average tensile strength of 20–30 MPa^27^. As in Table 3, the pure PCL film developed in this study showed an average tensile strength of 29.59 ± 1.22 MPa and elongation at break of 302.96 ± 46.54%. No significant change (*p* > 0.05) was observed in tensile strength until the addition of 3% GSE between pure PCL and PCL/GSE composite films, but significantly decreased (*p* ≤ 0.05) to 27.31 MPa in the PCL/GSE 5% film. This decrease might be due to the increased level of GSE contents in PCL matrix, because it is known that typical additives except for cross-linking agents decrease tensile strength and increase elongation of the film^28^.

**Table 3:** Mechanical properties of the pure PCL and PCL/GSE composite films.

Composite	Mechanical properties	
	Tensile strength (MPa)	Elongation at break (%)
Pure PCL	29.59 ± 1.22^B^	302.96 ± 46.54^A^
PCL/GSE 1%	28.31 ± 1.08^AB^	334.87 ± 20.71^AB^
PCL/GSE 3%	27.99 ± 1.73^AB^	360.96 ± 25.62^AB^
PCL/GSE 5%	27.31 ± 2.27^A^	360.53 ± 15.13^B^

PCL: polycaprolactone; GSE: grapefruit seed extract.

Data are expressed as the mean ± standard deviation of five replicates.

The different uppercase letters (A and B) in the same column indicate a significant difference (*p* ≤ 0.05), as assessed by Duncan’s multiple range test.

On the other hand, the elongation at break gradually improved as the amount of GSE increased. The incorporation of 1, 3, and 5% GSE into the PCL matrix significantly increased (*p* ≤ 0.05) its elongation value from 302.96 ± 46.54 to 334.87 ± 20.71, 360.96 ± 25.62, and 360.53 ± 15.13%, respectively. A previous report has shown comparable results of improvement in elongation at break due to addition of GSE into the polymer matrix. This phenomenon can be attributed to glycerol components in GSE that usually act as plasticizers^29^. The increase of elongation at break in PCL/GSE composite films could be assumed to advance their flexibility, suggesting that they have the potential to be used in flexible packaging materials.

### *In vitro* antimicrobial activity of PCL/GSE composite films against *L*. *monocytogenes*

**Figure 4:**
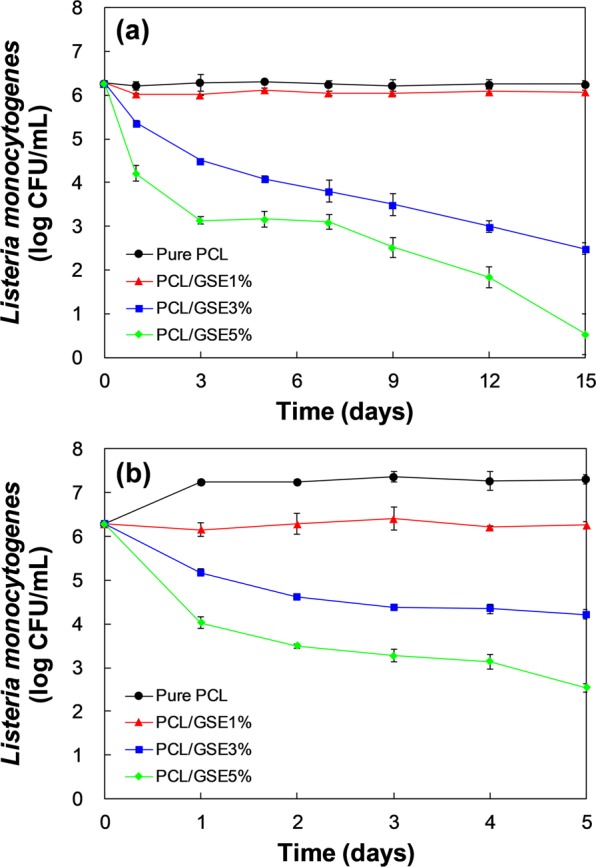
*In-vitro* anti-listerial effect of pure PCL and PCL/GSE composite films at (**a**) 4 and (**b**) 25 °C. PCL: polycaprolactone; GSE: grapefruit seed extract.

Figure 4 shows the *in vitro* anti-listerial effect of pure PCL and PCL/GSE composite films at 4 (Fig. 4a) and 25 °C (Fig. 4b). There were decreasing tendencies of *L*. *monocytogenes* counts in the PCL/GSE 3 and 5% treatment both in 4 and 25 °C storage groups throughout the storage time.

In the 4 °C storage group (Fig. 4a), the *L*. *monocytogenes* counts started from 6.28 ± 0.04 log CFU/mL and reached 6.26 ± 0.07, 6.07 ± 0.04, 2.49 ± 0.13, and 0.53 ± 0.47 log CFU/mL at 15 days in the pure PCL, PCL/GSE 1, 3, and 5% respectively. Specifically, the number of bacterial cells in the pure PCL film group was constant in the 4 °C group while the number increased approximately 1-log in the 25 °C group. This phenomenon was because of the lag phase of *L*. *monocytogenes*. The refrigeration temperature retarded the growth of *L*. *monocytogenes* and caused the *L*. *monocytogenes* to stay in the lag phase. Therefore, during the storage period, *L*. *monocytogenes* did not grow. However, if the storage period had been set longer than 15 days, we could have observed *L*. *monocytogenes* growth at the exponential phase after the lag phase. The PCL/GSE 1% film group also showed a similar tendency to the pure PCL film group. That is, the PCL/GSE 1% film had no significant antimicrobial activity against *L*. *monocytogenes*. Meanwhile, the PCL/GSE 3% and 5% film groups indicated a significant reduction in populations of *L*. *monocytogenes* showing 3.8- and 5.8-log reduction after 15 days, respectively. These results suggested that the PCL/GSE 3% and 5% films could be adequately used as antimicrobial packaging for refrigerated foods.

Figure 4b indicates the changes in bacterial cell numbers in the 25 °C storage group. At 25 °C, the population of *L*. *monocytogenes* changed from 6.28 ± 0.04 log CFU/mL to 7.31 ± 0.12, 6.26 ± 0.09, 4.22 ± 0.10, and 2.54 ± 0.09 log CFU/mL at 5 days in the pure PCL, PCL/GSE 1, 3, and 5% respectively. The number of *L*. *monocytogenes* in the pure PCL film-immersed solution gradually increased and remained constant until day 5 exhibiting no antibacterial activity. An inhibitory effect against *L*. *monocytogenes* was observed in the PCL/GSE 1% film-treated group in that the number of bacterial cells was not increased but was maintained in its initial state. The PCL/GSE 3% film group showed the 2.1-log reduction in bacterial cell number, indicating a strong bactericidal effect than that of PCL/GSE 1%. Further, in the PCL/GSE 5% film group, more rapid and stronger antibacterial effects were observed, with 3.7-log reduction in bacterial cell count within 5 days.

The antimicrobial effect seen in our results was better than that reported in a previous study which involved polylactic acid (PLA)/GSE and low-density polyethylene (LDPE)/GSE composite films^30^. This is presumed to be due to loss of GSE activity during the high thermal processing temperature used in the above studies. Melting temperature of PLA and LDPE are around 180 and 110 °C, respectively^31,32^. It implies that PCL, which can be used under relatively low thermal processing conditions, is a desirable candidate polymer retaining the inherent functions of GSE.

### Application of PCL/GSE composite films in commercial cheddar cheese packaging

**Figure 5:**
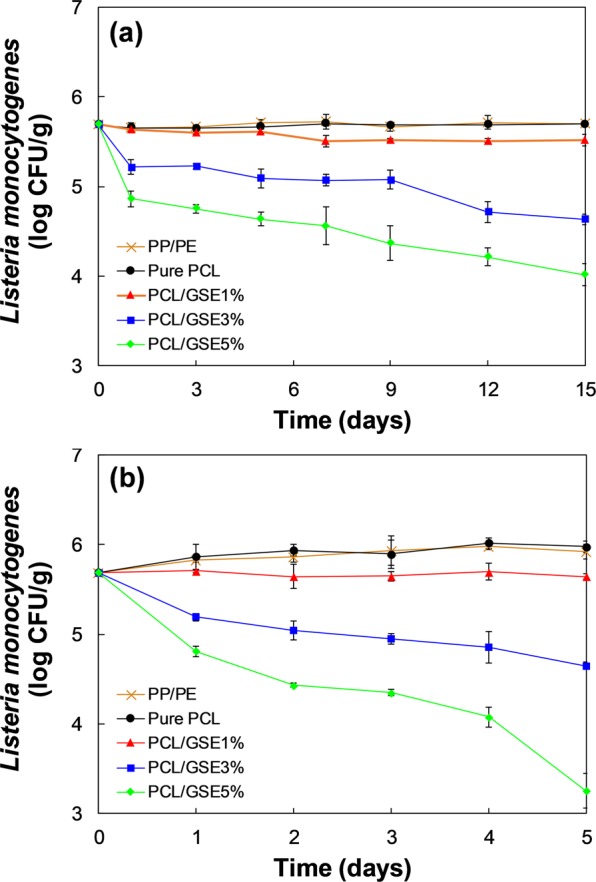
*Listeria monocytogenes* counts of cheddar cheese packaged in pure PCL, PP/PE (commercial cheese packaging film), and PCL/GSE composite films during storage at (**a**) 4 and (**b**) 25 °C. PP: polypropylene; PE: polyethylene; PCL: polycaprolactone; GSE: grapefruit seed extract.

Figure 5 represents the antibacterial effect of the PCL/GSE composite packaging films on cheddar cheese (Seoul Dairy Cooperative, Seoul, Korea) inoculated with *L*. *monocytogenes* during storage at 4 (Fig. 5a) and 25 °C (Fig. 5b). Both the commercial cheese packaging film [polypropylene (PP)/polyethylene (PE)] and the pure PCL film were considered control groups. The initial number of *L*. *monocytogenes* was 5.69 ± 0.02 log CFU/g.

In the 4 °C storage group (Fig. 5a), the populations of *L*. *monocytogenes* counts after 15 days were 5.70 ± 0.03, 5.70 ± 0.02, 5.52 ± 0.06, 4.64 ± 0.05, and 4.02 ± 0.12, in the PP/PE, pure PCL, PCL/GSE 1, 3, and 5%, respectively. The number of bacterial cells in the two control groups, PP/PE and pure PCL film groups, were slightly lower than that in the initial state. On the other hand, PCL/GSE composite films indicated outstanding antimicrobial activity. Significantly, the PP/PE and pure PCL film groups showed no increase in the population of *L*. *monocytogenes* throughout the storage time. This phenomenon was probably due to the effect of acidity regulator in commercial cheddar cheese for preservatives. Therefore, the PP/PE and pure PCL film groups maintained the initial number of *L*. *monocytogenes* after 15 days.

In the 25 °C storage group (Fig. 5b), the populations of *L*. *monocytogenes* counts after 5 days were 5.93 ± 0.08, 5.98 ± 0.06, 5.65 ± 0.03, 4.65 ± 0.04, and 3.25 ± 0.19, in the PP/PE, pure PCL, PCL/GSE 1, 3, and 5%, respectively. The number of bacterial cells in the two control groups increased only 0.2- and 0.3-log during storage period, and there was no significant difference (*p* > 0.05) was found between the two groups over the entire storage time. Although the number of bacteria in the control groups increased, it was lower than that observed *in vitro* test. This might be due to the fact that the various components of cheese, including acidity regulator and salt retarded the growth of *L*. *monocytogenes* to some extent. The PCL/GSE 1% group showed retention of the initial number of bacteria, while the PCL/GSE 3% and 5% groups indicated the decrease in the bacterial counts. Specifically, the PCL/GSE 5% film group represented the marked antimicrobial activity. The PCL/GSE 5% film caused the 2.4-log reduction in bacterial cell number in the cheese samples after 5 days.

Collectively, compared with 25 °C room temperature (RT) conditions, the storage in the 4 °C refrigeration conditions was done for a relatively longer period of time, but the bactericidal effect was less pronounced. This is because the rate at which the active ingredient acts was slower in refrigerated conditions than at RT. Further, the inhibitory effect of the PCL/GSE composite film was less in cheddar cheese than in broth conditions. This is in agreement with the result of a previous study showing that such discrepancies could be a result of the complexity of the real food system^33^.

### Biodegradability in soil of PCL/GSE composite films

#### Weight loss of PCL/GSE composite films

**Figure 6:**
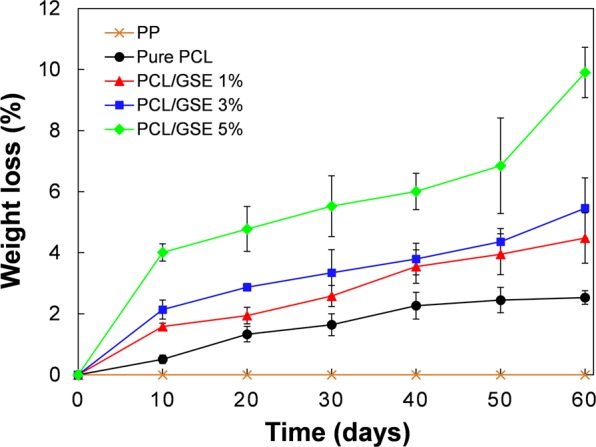
Weight loss of soil-buried pure PCL and PCL/GSE composite films at 30 °C. PP: polypropylene; PCL: polycaprolactone; GSE: grapefruit seed extract.

Biodegradability of the pure PCL and PCL composite films was analyzed via soil burial testing. Biodegradation was assessed based on weight loss at 10-day intervals from days 10 to 90 (Fig. 6). PP film, a non-biodegradable polymer, was used as a negative control. The biodegradability rate was distinctly different in each group. The weight loss rate of the pure PCL film was slowest during the entire study period, at only 2.5%. The biodegradability was faster as the incorporated amount of GSE increased. The weight loss of the PCL/GSE 5% composite film sharply increased during 10 days, showing 5% loss, which continuously increased to 10% by day 60. The order of increment in the rate of biodegradation was PCL/GSE 5% > PCL/GSE 3% > PCL/GSE 1% > pure PCL > PP. The crystallinity is one of the factors effective in biodegradation of polymers. It is known that the amorphous phase of the polymers is easy to degrade compared with crystal phase^34,35^. The results of the XRD analysis in this study showed that the crystallinity in PCL/GSE composite films was decreased as GSE content increased. Therefore, the rate of biodegradation was faster in the PCL/GSE 5% composite film group probably because of its relatively low crystallinity compared to that of the pure PCL film. The weight change was increased proportionally with increasing content of GSE added as a functional ingredient during the storage period. In the PP film used as a negative control, no weight change was observed until day 60 (biodegradation not occurred). These results indicate that addition of GSE facilitated biodegradation of the composite films.

#### Morphology of PCL/GSE composite films

**Figure 7:**
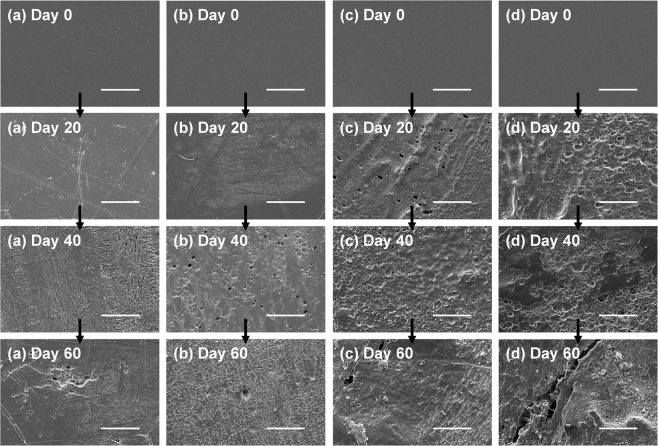
SEM micrographs (magnification × 500) of surface of the (**a**) pure PCL, (**b**) PCL/GSE 1%, (**c**) PCL/GSE 3%, and (**d**) PCL/GSE 5% before and after biodegradation at 30 °C. Scale bar is 100 μm. PCL: polycaprolactone; GSE: grapefruit seed extract.

The surface morphology of the samples was evaluated via scanning electronic microscopy (SEM) to investigate biodegradability in soil burial conditions over 60 days (Fig. 7). In general, the initial state of each sample had a relatively smooth surface. After 20 days, some scratches were observed on part of the surface of the pure PCL film samples. In the PCL/GSE 5% composite film group, surface degradation and formation of holes were clearly observed. With increasing burial time, the biodegradation pattern was more evident on the film surface in all experimental groups. After 40 days, most of the composite film surface was found to be degraded, as evidenced by scratches and holes. In the pure PCL film, there were no holes occurring for 40 days while many holes were observed throughout the surface in the PCL/GSE 5% film. After 60 days, severe scratches and cracks were observed over most of the surface in the pure PCL film group, and the surface structure of the PCL/GSE 5% film was found to have severely collapsed. The biodegradation rate of the samples increased with GSE concentration, similar to the weight loss findings. Thus, it was found that the low crystallinity of the polymer accelerated the breakdown of the polymer matrix, leading to increased degradation.

## Conclusion

In the present study, the eco-friendly PCL films with functionality and biodegradability were developed by incorporating GSE as an antimicrobial agent using a twin-screw extrusion process. Various characteristics structural, colorimetric, thermal, and mechanical properties were analyzed. Antimicrobial activity against *L*. *monocytogenes*, a gram-positive bacteria associated with food poisoning, was also tested. Biodegradability of the composites films was verified by examining surface and weight changes of the film samples after storage for 60 days at composting conditions of 30 °C. The PCL/GSE composite films showed high antibacterial activity against target bacteria while maintaining the inherent thermal and mechanical properties of PCL. In addition, significant weight loss and surface degradation were observed during storage. In summary, the present study developed the PCL/GSE composite films and showed their potential use as an alternative to non-biodegradable food packaging materials.

## Methods

### Materials

PCL (Mw = 70,000–90,000) was obtained from Sigma-Aldrich Co., Ltd. (St Louis, MO, USA). GSE (DF-100; Quinabra-Quimica Natural Brasileira Ltd., Brazil) was purchased from FA Bank Co. (Anseong, Korea). According to the manufacturer, the extract comprised 50% glycerol, 49.49% pure GSE, and 0.51% naringin.

### Preparation of PCL/GSE composite films

Biodegradable PCL/GSE composite films were prepared by using a co-rotating twin-screw extruder (Haake Process 11; Thermo Fisher Scientific Inc., Karlsruhe, Germany) with a screw diameter of 11 mm and length of 440 mm.

PCL pellets and GSE at different concentrations (1, 3, and 5%, *w/w*) were mixed and fed into the feeder. To ensure homogeneous distribution of the GSE in PCL, pellets were extruded and pelletized three times before they were extruded in the form of a sheet. The strand die (2.0-mm diameter) and sheet die (30-mm width × 1.0-mm height) was used for producing pellets and sheets, respectively. The speed of the feeder and the screw was 5 and 50 rpm, respectively. The temperature profile was varied from 65 °C at the feeder to 75 °C at the die.

The extruded sheets (thickness of 1.0 ± 0.05 mm) were thinned to films using a heating press machine (Ocean Science Co., Uiwang, Korea) for analyzing the film properties. Compression was carried out between stainless steel plates for 2 min at 40 MPa and 59 °C. The heated plates were removed from the machine and immediately cooled to RT. Films were then obtained with a thickness of 0.12 ± 0.01 mm and kept in a thermo-hygrostat (Labmade 011; Sejong Technology Co., Ltd., Bucheon, Korea) at 25 °C and 50% relative humidity before being tested.

### Characterization of PCL/GSE composite films

#### FT-IR spectroscopic analysis

The FT-IR spectra of the pure PCL and the PCL/GSE composite films were obtained using an FT-IR spectrometer (Cary 630; Agilent Technologies, Inc., Santa Clara, CA, USA) in the infrared range of 4000–500 cm^−1^. Spectra were collected in 128 scans at a resolution of 4 cm^−1^ for each sample.

#### Color measurement

The surface color of the pure PCL and PCL/GSE composite films was measured using a colorimeter (CR-400; Konica Minolta Sensing Inc., Osaka, Japan) calibrated with a standard white plate (*Y* = 93.8, *x* = 0.3131, and *y* = 0.3191). The results were described per the CIE L^*^a^*^b^*^ system parameters; L^*^ (lightness), a^*^ (red to green), and b^*^ (yellow to blue). Each film was measured at five different locations in triplicate. The total color difference (∆E^*^) was calculated using the following Eq. (1).

1 $$\Delta {E}^{\ast }=\sqrt{{(\Delta {L}^{\ast })}^{2}+{(\Delta {a}^{\ast })}^{2}+{(\Delta {b}^{\ast })}^{2}}$$

where $$\Delta {L}^{\ast }={L}_{sample}^{\ast }-{L}_{control}^{\ast }$$, $$\Delta {a}^{\ast }={a}_{sample}^{\ast }-{a}_{control}^{\ast }$$, and $$\Delta {b}^{\ast }={b}_{sample}^{\ast }-{b}_{control}^{\ast }$$.

#### DSC analysis

Thermal properties of the pure PCL and PCL/GSE composite films were analyzed using DSC and TGA. DSC was measured using a differential scanning calorimeter (Q20; TA Instruments Inc., New Castle, DE, USA) under a nitrogen atmosphere. Film samples (5 mg) were hermetically sealed in an aluminum pan and an empty hermetic pan was used as a reference. Samples were heated from 25 to 100 °C with a heating rate of 10 °C/min. The thermal parameters such as *T*_m_ and *H*_m_ of the film samples were obtained from the DSC thermograms. The *X*_c_ of the materials was calculated using the following Eq. (2). The measurements were repeated in triplicate.

2 $${X}_{c}( \% )=(\Delta {H}_{m}/\Delta {H}_{m}^{^\circ })\times 100$$

in which ∆*H*_m_ and ∆*H*^*°*^_m_ are the enthalpy for the melting process and melting enthalpy of 100% crystalline PCL (136.1 J/g)^36^, respectively.

#### TGA test

TGA was performed to investigate thermal stabilities of the pure PCL and PCL/GSE composite films by using a thermogravimetric analyzer (Q500; TA Instruments Inc., New Castle, DE, USA). Samples were heated from 25 to 700 °C and held at an isothermal temperature for 3 min. The temperature was raised at a constant rate of 10 °C/min under nitrogen atmosphere. The results were plotted as weight loss versus temperature.

#### XRD analysis

XRD analysis for the pure PCL and PCL/GSE composite films was carried out using an X-ray diffractometer (SmartLab; Rigaku Co., Tokyo, Japan) with Cu Kα radiation at a current of 200 mA. The XRD spectra were obtained by scanning in the range of 2θ = 15–25° at a scanning rate of 4 °C/min at an accelerating voltage of 45 kV.

#### Measurement of mechanical properties

Mechanical properties including tensile strength and elongation at break of the pure PCL and PCL/GSE composite films were measured following the ASTM D882-02 standard method using a universal testing machine (Instron 4467; Instron Engineering Corp., Norwood, MA, USA). Each sample was cut into a size of 15 × 100 mm and placed between the grip heads of the machine. The cross-head speed was set to 500 mm/min, and the initial gauge length and grip separation were 50 mm. Each sample was analyzed at least five times.

### *In vitro* and *in vivo* antimicrobial activity tests of PCL/GSE composite films against *L*. *monocytogenes*

#### Inoculum preparation

*Listeria monocytogenes* strain (ATCC 19115) was obtained from the American Type Culture Collection (ATCC). For experimental use, the *L*. *monocytogenes* strain was aerobically grown with shaking in tryptic soy broth (Becton, Dickinson and Co., Sparks, MD, USA) at 37 °C prior to use. After cultivation, the cells were diluted to the target concentration for inoculation.

#### *In vitro* antimicrobial activity test

The film samples (3 × 3 cm²) were immersed into the previously prepared 10-mL bacterial suspension (10^6^ CFU/mL of *L*. *monocytogenes*). Samples were stored at 4 °C (a storage temperature for commercial cheese products) for 15 days and at 25 °C for 5 days. Aliquots of the bacterial cells were taken and diluted. The number of bacterial cells during storage was evaluated. Bacterial counts were determined in triplicate by plating the cell suspensions on Oxford agar (MBO1310; Kisan Bio Co., Ltd., Seoul, Korea). Colonies were counted after 48 h of incubation at 37 °C, and microbial counts were determined as the log of CFU per mL of samples.

#### *In vivo* antimicrobial activity test (Application of PCL/GSE composite films in commercial cheddar cheese packaging)

The *L*. *monocytogenes* strains were grown in tryptic soy broth medium (Becton, Dickinson and Co.) with shaking at 37 °C. Commercial cheddar cheese was used as a model food to test the antimicrobial property of the PCL/GSE composite films. Both the PP/PE and pure PCL films were considered control groups. Each cheese sample was cut into pieces of 3 × 3 cm². A 50-μl aliquot of bacterial suspension (10^8^ CFU/mL) was spread evenly on the surfaces of cheddar cheese followed by air-drying in a laminar flow clean-bench. Identical sized film pieces were contacted with contaminated food samples. Samples were stored at 4 °C for 15 days and at 25 °C for 5 days. Changes in the numbers of *L*. *monocytogenes* on the surfaces of cheddar cheese treated with the PP/PE, pure PCL and PCL/GSE composite films were determined during the storage period. On each day, one stored cheddar cheese sample (2.25 g) was placed into a Whirl-Pak bag (BO1341WA; Nasco, Fort Atkinson, WI, USA) containing 22.5 mL of PBS buffer (10%, *w/v*) and blended for 3 min using a stomacher (HG 400 V; Mayo International srl, Novate Milanese, Italy). The diluted aliquots were spread on Oxford agar. The agar plates were incubated at 37 °C for 48 h. Microbial counts were determined in triplicate and expressed as the log of CUF per g of samples.

### Measurement of biodegradability in soil (soil burial test) of PCL/GSE composite films

#### Weight loss of PCL/GSE composite films

Soil burial testing was conducted to examine biodegradability of the pure PCL and PCL/GSE composite films. PP film, a non-biodegradable polymer, was used as a negative control. Square-shaped film samples (2 × 2 cm2) were buried in commercial compost soil. The specimens were incubated at an environmental temperature of 30 °C in a closed chamber. Film samples were taken out from the compost soil after incubation and washed with distilled water followed by drying in a desiccator to a constant weight. Five specimens were analyzed 10-day intervals from days 10 to 90. Weight loss of the samples was calculated using Eq. (3) below:

3 $${\rm{Weight}}\,{\rm{loss}}\,( \% )=[({W}_{1}-{W}_{2})/{W}_{1}]\times 100$$

where *W*_1_ and *W*_2_ represent weight of dried film samples before and after the compost soil burial, respectively.

#### Morphology of PCL/GSE composite films

The surface morphology of the pure PCL and PCL/GSE composite films was examined using SEM (FEI Quanta FEG 250; FEI Technologies Inc., Hillsboro, OR, USA) evaluating its biodegradability. The surface analysis was carried out under an acceleration voltage of 5 kV and a magnification of ×500.

### Statistical analysis

All experiments, except TGA test and XRD analysis, which were performed one time, were carried out at least in triplicate. Data analyses were carried out using the statistical analysis system (SAS) software version 9.3 (SAS Institute Inc., Cary, NC, USA). The significant differences (*p* ≤ 0.05) between result values in the samples in the measurements of their color properties (L^*^, a^*^, b^*^, and ΔE^*^), thermal properties (*T*_m_, Δ*H*_m_, and *X*_c_), mechanical properties (tensile strength and elongation at break), antimicrobial activities (*in vitro* and *in vivo*), and biodegradability in soil were identified one-way analysis (ANOVA) followed by Duncan’s multiple range tests.

## Data Availability

The data sets generated during the current study are available from the corresponding author on reasonable request.
